# Spatiotemporal distribution of malaria and the association between its epidemic and climate factors in Hainan, China

**DOI:** 10.1186/1475-2875-9-185

**Published:** 2010-06-25

**Authors:** Dan Xiao, Yong Long, Shanqing Wang, Liqun Fang, Dezhong Xu, Guangze Wang, Lang Li, Wuchun Cao, Yongping Yan

**Affiliations:** 1 Department of Epidemiology, Faculty of Preventive Medicine, Fourth Military Medical University, Xi'an, China; 2 Hainan Center for Disease Control and Prevention, Haikou, China; 3 Beijing Institute of Microbiology and Epidemiology, State Key Laboratory of Pathogen and Biosecurity, Beijing, China

## Abstract

**Background:**

Hainan is one of the provinces most severely affected by malaria epidemics in China. The distribution pattern and major determinant climate factors of malaria in this region have remained obscure, making it difficult to target countermeasures for malaria surveillance and control. This study detected the spatiotemporal distribution of malaria and explored the association between malaria epidemics and climate factors in Hainan.

**Methods:**

The cumulative and annual malaria incidences of each county were calculated and mapped from 1995 to 2008 to show the spatial distribution of malaria in Hainan. The annual and monthly cumulative malaria incidences of the province between 1995 and 2008 were calculated and plotted to observe the annual and seasonal fluctuation. The Cochran-Armitage trend test was employed to explore the temporal trends in the annual malaria incidences. Cross correlation and autocorrelation analyses were performed to detect the lagged effect of climate factors on malaria transmission and the auto correlation of malaria incidence. A multivariate time series analysis was conducted to construct a model of climate factors to explore the association between malaria epidemics and climate factors.

**Results:**

The highest malaria incidences were mainly distributed in the central-south counties of the province. A fluctuating but distinctly declining temporal trend of annual malaria incidences was identified (Cochran-Armitage trend test *Z *= -25.14, *P *< 0.05). The peak incidence period was May to October when nearly 70% of annual malaria cases were reported. The mean temperature of the previous month, of the previous two months and the number of cases during the previous month were included in the model. The model effectively explained the association between malaria epidemics and climate factors (*F *= 85.06, *P *< 0.05, adjusted *R *^2 ^= 0.81). The autocorrelations of the fitting residuals were not significant (*P *> 0.05), indicating that the model extracted information sufficiently. There was no significant difference between the monthly predicted value and the actual value (*t *= -1.91, *P *= 0.08). The *R *^2 ^for predicting was 0.70, and the autocorrelations of the predictive residuals were not significant (*P *> 0.05), indicating that the model had a good predictive ability.

**Discussion:**

Public health resource allocations should focus on the areas and months with the highest malaria risk in Hainan. Malaria epidemics can be accurately predicted by monitoring the fluctuations of the mean temperature of the previous month and of the previous two months in the area. Therefore, targeted countermeasures can be taken ahead of time, which will make malaria surveillance and control in Hainan more effective and simpler. This model was constructed using relatively long-term data and had a good fit and predictive validity, making the results more reliable than the previous report.

**Conclusions:**

The spatiotemporal distribution of malaria in Hainan varied in different areas and during different years. The monthly trends in the malaria epidemics in Hainan could be predicted effectively by using the multivariate time series model. This model will make malaria surveillance simpler and the control of malaria more targeted in Hainan.

## Background

In 2006, there were 247 million malaria cases among the 3.3 billion people living under the threat of malaria worldwide, which resulted in over a million deaths [1]. Malaria is a crucial public health problem in China. In 2007, there were 46,988 cases and 18 deaths reported in 1,097 Chinese counties [2]. In 2004, the Hainan province was severely affected by malaria epidemics with 24.4% of the malaria cases and 35.3% of the falciparum malaria cases in China [3]. Although the number of malaria cases decreased from 5,864 to 1,600 during the late 1990s, epidemic fluctuations were still reported at the beginning of this century in Hainan [4].

Previous studies have examined malarial distribution patterns around the world, including the global distribution of malaria [5], population at risk [5], and the global distribution of falciparum malaria [6,7]. Other studies have investigated malarial distribution patterns in China, particularly in provinces with high malaria risks. For instance, studies have examined the geographic distributions, demographic patterns and temporal trends of falciparum malaria in China [8], spatial distribution and clustering of malaria in Anhui [9], and spatiotemporal distribution pattern of malaria in Yunnan [10]. However, malaria epidemics vary in different spatial and temporal levels. Hainan has the second highest malaria incidence in China. The spatial distribution of malaria was previously reported for Hainan for the years 1995 to 1999 [11], but since then, the incidence has fluctuated, and little is known about malaria epidemics since 2000.

The influence of climate factors on malaria epidemics has been controversial. Some researchers have reported that climate factors played an important role in the emergence and re-emergence of malaria epidemics in different regions around the world [12-17]. In contrast, other studies maintained that associations between the regional changes in climate and local malaria resurgences were questionable [18,19]. According to Patz and Olson, this debate may in part be due to the varying quality of long-term disease data, as well as the difficulty controlling the sociodemographic and biological data [20]. Wen et al. constructed a linear model that would allow climate factors to predict malaria epidemics in Hainan by using reliable quality malaria data provided by the Hainan Center for Disease Control and Prevention [21]. However, since the researchers assumed a simple linear correlation existed between malaria epidemics and climate factors without testing the linearity of the relationship, there remain uncertainties about their conclusions. Therefore, it is necessary to perform further studies to establish the relationship between malaria epidemics and climate factors in Hainan using high-quality data and reliable methods.

This study evaluated the spatiotemporal distribution of malaria in Hainan from 1995 to 2008. Using data from this period, a model that describes the association between malaria epidemics and climate factors in Hainan was constructed using geographic information system (GIS)-based spatial analysis and multivariable time series analysis. The aim of this study is to provide a scientific basis for malaria surveillance and control in the Hainan province.

## Methods

### Study area

The study area was the main island of the Hainan province, which is located at north latitude 18°10' - 20°10' and east longitude 108°37' - 111°03', in southern China. The region has an area of 33.9 thousand square kilometers and a population of 8.26 million [22]. The province is characterized by mountainous, hilly landscape features and different latitudinal landscapes. The tropical monsoon and tropical marine climate together produce high temperatures and rich rainfall, which are suitable for malaria transmission.

### Data collection and management

The malaria cases records were obtained from the Hainan Center for Disease Control and Prevention (CDC). The malaria cases were diagnosed in the medical and health units of each county and reported to the Hainan CDC through the Reporting System of Communicable Diseases and Unexpected Public Health Events. The population of each county from 1995 to 2008 was derived from the Hainan statistical yearbooks, which were compiled by the Hainan Provincial Bureau of Statistics. The demographic data in the yearbooks were collected from annual statistical reports from each county, which were obtained from surveys. The incidences of malaria cases for each county were geo-coded and matched to the corresponding polygon on a digital map of Hainan.

For the years 1995 to 2008, monthly climate data from approximately 700 surveillance stations in mainland China were collected from the China Meteorological Data Sharing Service System, which was organized by the China National Meteorological Information Centre (CNMIC) [23]. The CNMIC processes and publishes the meteorological observation data submitted monthly by meteorological bureaus in each province, region and municipality according to a uniform protocol. To create the monthly climate surfaces of mean temperature, mean maximum temperature, mean minimum temperature, accumulative rainfall, and mean relative humidity in China, models were designed using the data from the surveillance stations. Then parameter estimates were performed followed by spatial interpolation using the ordinary kriging method. The monthly, county-level estimates of the climate variables (spatial means) from 1995 to 2008 were then extracted using the spatial analyst model with ArcGIS 9.2 software by overlapping the vector county map of Hainan on the raster maps (ArcGIS 9.2, Environmental Systems Research Institute, Redlands, CA, USA).

### Spatial distribution analysis

All counties were divided into six regions. The cumulative and annual malaria incidences in each county were mapped from 1995 to 2008. Each region was marked with a different color on the county-level digital map.

### Temporal distribution analysis

The annual malaria incidences from 1995 to 2008 were calculated and plotted to observe annual fluctuations in Hainan. The Cochran-Armitage trend test was employed to examine temporal trends in the annual malaria incidence. Cumulative malaria incidences for each month from 1995 to 2000 and from 2001 to 2008 were calculated and plotted to observe seasonal fluctuations.

### Cross correlation and autocorrelation analysis

A cross correlation analysis was conducted to detect the effects of climate factors on malaria transmission with a lag time of six months. The cross correlation could be observed if the cross correlation coefficient (*CCF*) was larger than two times the standard error (*SE*). An autocorrelation analysis was performed to explore whether the monthly malaria incidence was affected by the incidence in previous months using the Ljung-Box Q test, autocorrelation coefficient (AC) and partial autocorrelation coefficient (PAC).

### Multivariate time series analysis

Due to the lack of climate information for 2008, the model was constructed using data from 1995 to 2006, and the number of malaria cases in each month in 2007 was predicted to evaluate the effect of the model. The variables that significantly correlated with the malaria incidence were added into the model for variable selection. Spearman's rank correlation coefficient and co-linearity statistics were calculated to examine the co-linearity between the variables added to the model for variable selection. Both analyses were carried out using SPSS 16.0 (SPSS Inc., Chicago, IL).

Based on the above analyses, the Poisson model was constructed as follows:

where

and *N*_*t *_is the number of cases in year *t*, and *ε *represents random noise. The term  denotes the effect of climate factors, and  describes the effect of auto regression. Parameters *m*, *p *and *q *represent the number of climate variables, the maximum number of months of previous climate variables, and the maximum number of months of previous cases that were included in the model, respectively. The term *x*_*t-k *_represents the climate factors, including the mean temperature, mean maximum temperature, mean minimum temperature, accumulative rainfall and mean relative humidity of each month. The relationship between *y*_*i(t-k*) _and *x*_*t-k *_was evaluated using scatter plots of monthly malaria incidence and monthly climate factors. The term *α*_0 _is a constant term, and *α*_*i*_, *β*_*i *_and  are coefficients for the variables. The model was adjusted by the provincial population each year to avoid population offset. The stepwise least squares method was employed to fit the model. The *F *test, adjusted *R *^2 ^of fitting, and Ljung-Box Q test for autocorrelation of fitting residuals were conducted to test the fitting validity of the model. The paired t-test was used to determine whether each predictive value was significantly different from the actual one. The *R *^2 ^for predicting and the Ljung-Box Q test for autocorrelation of predictive residuals were conducted to test the predictive ability of the model.

## Results

### Spatial distribution of malaria in Hainan

**Figure 1:**
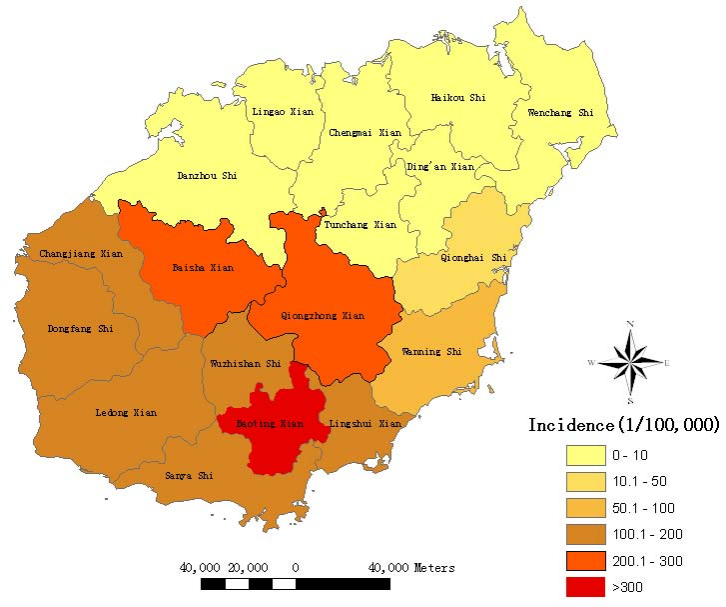
**Cumulative malaria incidence at the county level in Hainan, China, 1995-2008**. The counties are color coded according to the malaria incidence. The highest malaria incidences were mainly distributed in the central-south counties of the province.

**Figure 2:**
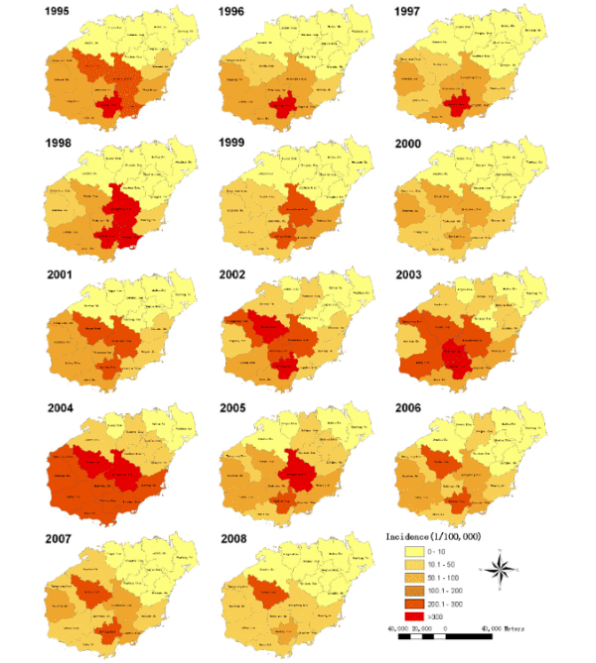
**Annual malaria incidence at the county level in Hainan, China, 1995-2008**. The counties are color coded according to the malaria incidence. The highest malaria incidence during this period was not restricted to a single county.

There were 64,478 reported malaria cases in Hainan from 1995 to 2008. Malaria cases were reported in all counties of the province during this period, with incidences ranging from 1.24 to 313.68 per 100,000. The highest malaria incidences were mainly distributed in the central-south counties of the province, such as Baoting, Baisha and Qiongzhong. Additionally, serious malaria epidemics also occurred in southern counties, such as Wuzhishan, Lingshui, Changjiang and Ledong (Figures 1 and 2).

### Temporal distribution of malaria in Hainan

**Figure 3:**
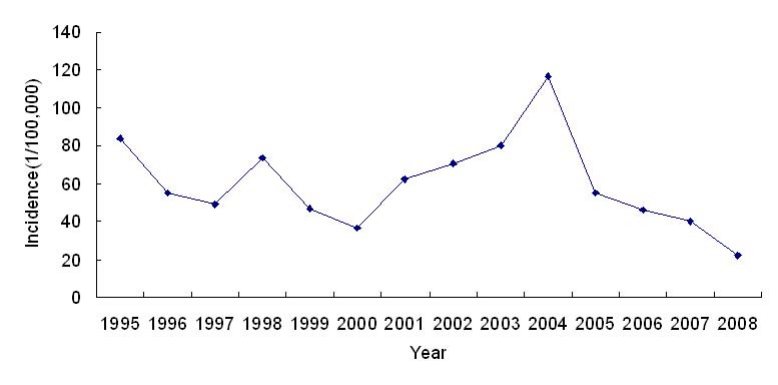
**Annual malaria incidence in Hainan, China, 1995-2008**. The highest malaria incidence was 116.39 per 100,000 in 2004 and the lowest incidence was 22.06 per 100,000 in 2008. A fluctuating but distinctly declining temporal trend of annual malaria incidence is notable.

**Figure 4:**
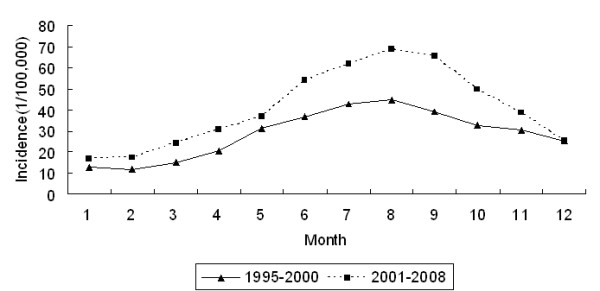
**Monthly malaria incidence during different time periods in Hainan, China**. The peak period occurred between May and October, during which nearly 70% of the annual malaria cases were reported. The month with the lowest malaria incidence was February during the 1995-2000 period and January during the 2001-2008 period, whereas the highest malaria incidence was reported during the month of August for both of these periods.

The annual malaria incidence in Hainan varied from 1995 to 2008. The highest incidence of 116.39 cases per 100,000 occurred in 2004, and the lowest incidence of 22.06 cases per 100,000 occurred in 2008. A fluctuating but distinctly declining temporal trend of annual malaria incidence was identified (Figure 3, Cochran-Armitage trend test *Z *= -25.14, *P *< 0.05). Malaria cases were reported every month, and the peak period occurred between May and October, during which nearly 70% of the annual malaria cases were reported. The month with the lowest malaria incidence was February during the 1995-2000 period and January during the 2001-2008 period, whereas the highest malaria incidence was reported during the month of August for both of these periods (Figure 4).

### Correlation between monthly malaria incidence and climate factors

As shown in Table 1, the monthly malaria incidence in Hainan was positively correlated with climate factors in the same month, including the mean temperature (MT _0_), mean maximum temperature (MaxT _0_), mean minimum temperature (MinT _0_) and accumulative rainfall (R _0_). Additionally, the monthly malaria incidence was positively correlated with climate factors recorded during the previous month, including the mean temperature (MT _1_), mean maximum temperature (MaxT _1_), mean minimum temperature (MinT _1_), and accumulative rainfall (R _1_). Moreover, the monthly malaria incidence was positively correlated with climate factors during the previous two months, including the mean temperature (MT _2_), mean maximum temperature (MaxT _2_), mean minimum temperature (MinT _2_) and accumulative rainfall (R _2_), as well as with climate factors from the previous three months, including the mean temperature (MT _3_), mean maximum temperature (MaxT _3_) and mean minimum temperature (MinT _3_). The correlation between the monthly malaria incidence and mean relative humidity was not significant. Therefore, this variable was not included in the model.

**Table 1:** Cross correlation between monthly malaria incidence and climate factors in Hainan, China

Lag	MT			MaxT			MinT			R			MH	
	
	*CCF*	*SE*		*CCF*	*SE*		*CCF*	*SE*		*CCF*	*SE*		*CCF*	*SE*
-6	-0.53	0.08		-0.47	0.08		-0.38	0.08		-0.54	0.08		0.02	0.08
-5	-0.30	0.08		-0.25	0.08		-0.20	0.08		-0.47	0.08		-0.02	0.08
-4	0.04	0.08		0.04	0.08		0.09	0.08		-0.31	0.08		-0.03	0.08
-3	0.34	0.08		0.28	0.08		0.34	0.08		-0.06	0.08		-0.01	0.08
-2	0.57	0.08		0.45	0.08		0.52	0.08		0.19	0.08		0.03	0.08
-1	0.65	0.08		0.48	0.08		0.59	0.08		0.36	0.08		0.09	0.08
0	0.55	0.08		0.37	0.08		0.49	0.08		0.42	0.08		0.08	0.08
1	0.30	0.08		0.15	0.08		0.26	0.08		0.35	0.08		0.02	0.08
2	-0.03	0.08		-0.12	0.08		-0.01	0.08		0.16	0.08		-0.04	0.08
3	-0.36	0.08		-0.38	0.08		-0.28	0.08		-0.10	0.08		-0.10	0.08
4	-0.60	0.08		-0.55	0.08		-0.46	0.08		-0.38	0.08		-0.16	0.08
5	-0.66	0.08		-0.57	0.08		-0.53	0.08		-0.49	0.08		-0.10	0.08
6	-0.56	0.08		-0.46	0.08		-0.45	0.08		-0.56	0.08		-0.13	0.08

*CCF*: cross correlation coefficient. *SE*: standard error. Lag: the number of lagged months. The cross-correlation of each lagged month between the monthly malaria incidence and climate factors could be identified if the *CCF *was larger than two times the *SE*.

### Autocorrelation of monthly malaria incidence

The P value of the Ljung-Box Q Statistic of each lagged month was less than 0.05. The absolute value of the autocorrelation coefficient and partial autocorrelation coefficient during the first three lagged months were greater than those of other lagged months, which indicated that there was a strong autocorrelation of monthly malaria incidence during the first three lagged months (Table 2).

**Table 2:** Autocorrelation and partial correlation of monthly malaria incidence, and the fitting and predictive residuals of the model

Lag	Monthly malaria incidence					Fitting residual					Predictive residual			
	
	*AC*	*PAC*	*LB*	*P*		*AC*	*PAC*	*LB*	*P*		*AC*	*PAC*	*LB*	*P*
1	0.84	0.84	102.48	< 0.01		-0.06	-0.06	0.20	0.66		0.29	0.29	1.25	0.26
2	0.60	-0.33	155.20	< 0.01		-0.04	-0.05	0.31	0.86		-0.33	-0.45	3.12	0.21
3	0.30	-0.33	168.51	< 0.01		0.06	0.06	0.54	0.91		-0.13	0.18	3.45	0.33
4	0.03	-0.07	168.64	< 0.01		0.03	0.03	0.59	0.97		0.17	0.01	4.04	0.40
5	-0.19	-0.05	173.85	< 0.01		0.04	0.05	0.67	0.99		0.07	-0.03	4.17	0.53
6	-0.27	0.17	185.02	< 0.01		0.01	0.02	0.68	0.99		-0.02	0.09	4.18	0.65
7	-0.24	0.12	194.14	< 0.01		0.05	0.06	0.88	0.99		-0.17	-0.26	5.11	0.65
8	-0.11	0.15	196.02	< 0.01		-0.04	-0.04	0.98	0.99		-0.28	-0.18	8.47	0.39
9	0.09	0.18	197.33	< 0.01		0.07	0.07	1.32	0.99		-0.19	-0.16	10.47	0.31
10	0.32	0.20	213.76	< 0.01		-0.14	-0.14	2.62	0.99		0.06	-0.02	10.80	0.37
11	0.51	0.08	254.12	< 0.01		0.06	0.06	2.92	0.99					
12	0.56	-0.18	303.34	< 0.01		-0.22	-0.25	6.51	0.89					

*AC*: autocorrelation coefficient. *PAC*: partial autocorrelation coefficient. *LB*: Ljung-Box Q Statistic. Lag: the number of lagged months. For the monthly malaria incidence, P < 0.05 indicates a strong autocorrelation of monthly malaria incidence. For the fitting and predictive residuals, P > 0.05 indicates that the model extracted the information sufficiently and had good prediction validity.

### Model evaluation and prediction

The variables MT _0_, MaxT _0_, MinT _0_, R _0_, MT _1_, MaxT _1_, MinT _1_, R _1_, MT _2_, MaxT _2_, MinT _2_, R _2_, MT _3_, MaxT _3_, MinT _3_, N _1_, N _2_, and N _3 _were added into the model for variable selection. There were 131 pairs of variables that correlated with each other significantly (Table 3, *P *< 0.05 or *P *< 0.01), and 12 variables with tolerance values lower than 0.1 and variance inflation factors higher than 10 (Table 4), which indicates the co-linearity of these variables. The following model gave the best results:

**Table 3:** Spearman's rank correlation coefficient between variables added into the model

	**MT**_**0**_	**MT**_**1**_	**MT**_**2**_	**MT**_**3**_	**MaxT**_**0**_	**MaxT**_**1**_	**MaxT**_**2**_	**MaxT**_**3**_	**MinT**_**0**_	**MinT**_**1**_	**MinT**_**2**_	**MinT**_**3**_	**R**_**0**_	**R**_**1**_	**R**_**2**_	**N**_**1**_	**N**_**2**_	**N**_**3**_
**MT**_**0**_	1.00																	
**MT**_**1**_	0.82**	1.00																
**MT**_**2**_	0.46**	0.82**	1.00															
**MT**_**3**_	-0.01	0.47**	0.82**	1.00														
**MaxT**_**0**_	0.77**	0.57**	0.24**	-0.14	1.00													
**MaxT**_**1**_	0.69**	0.76**	0.55**	0.23**	0.86**	1.00												
**MaxT**_**2**_	0.45**	0.69**	0.75**	0.55**	0.60**	0.86**	1.00											
**MaxT**_**3**_	0.10	0.45**	0.69**	0.75**	0.29**	0.59**	0.86**	1.00										
**MinT**_**0**_	0.89**	0.75**	0.45**	0.04	0.46**	0.41**	0.22**	-0.10	1.00									
**MinT**_**1**_	0.73**	0.89**	0.75**	0.46**	0.28**	0.44**	0.40**	0.22**	0.81**	1.00								
**MinT**_**2**_	0.41**	0.74**	0.89**	0.76**	-0.01	0.27**	0.44**	0.41**	0.53**	0.81**	1.00							
**MinT**_**3**_	-0.01	0.42**	0.74**	0.90**	-0.33**	-0.01	0.27**	0.45**	0.16	0.54**	0.81**	1.00						
**R**_**0**_	0.68**	0.79**	0.67**	0.35**	0.51**	0.67**	0.63**	0.39**	0.63**	0.69**	0.55**	0.27**	1.00					
**R**_**1**_	0.42**	0.68**	0.79**	0.67**	0.26**	0.49**	0.66**	0.63**	0.36**	0.63**	0.70**	0.57**	0.59**	1.00				
**R**_**2**_	0.03	0.43**	0.68**	0.79**	-0.07	0.25**	0.49**	0.66**	0.04	0.36**	0.64**	0.70**	0.36**	0.60**	1.00			
**N**_**1**_	0.33**	0.63**	0.76**	0.68**	0.13	0.39**	0.55**	0.55**	0.33**	0.58**	0.70**	0.63**	0.44**	0.58**	0.55**	1.00		
**N**_**2**_	-0.04	0.34**	0.63**	0.76**	-0.15	0.13	0.39**	0.55**	0.00	0.34**	0.59**	0.70**	0.20**	0.45**	0.59**	0.82**	1.00	
**N**_**3**_	-0.41**	-0.04	0.34**	0.63**	-0.41**	-0.15	0.13	0.40**	-0.36**	0.01	0.34**	0.59**	-0.15	0.21*	0.45**	0.55**	0.83**	1.00

*, *P *< 0.05. **, *P *< 0.01

**Table 4:** Co-linearity statistics of variables added into the model

Variables	Tolerance	VIF
**MT**_**0**_	0.02	51.02
**MT**_**1**_	0.02	25.26
**MT**_**2**_	0.02	47.61
**MT**_**3**_	0.03	39.79
**MaxT**_**0**_	0.04	24.24
**MaxT**_**1**_	0.03	38.42
**MaxT**_**2**_	0.03	37.78
**MaxT**_**3**_	0.05	22.01
**MinT**_**0**_	0.07	14.88
**MinT**_**1**_	0.06	15.71
**MinT**_**2**_	0.07	13.95
**MinT**_**3**_	0.07	14.49
**R**_**0**_	0.48	2.08
**R**_**1**_	0.41	2.43
**R**_**2**_	0.42	2.39
**N**_**1**_	0.17	5.77
**N**_**2**_	0.11	8.98
**N**_**3**_	0.17	5.93

*VIF*: variance inflation factors for individual variables. Tolerances and VIF are co-linearity statistics that measure the strength of the interrelationships among the variables incorporated into the model for variable selection. If a variable is very closely related to other variables, then the tolerance goes to 0, and the VIF gets very large.

**Figure 5:**
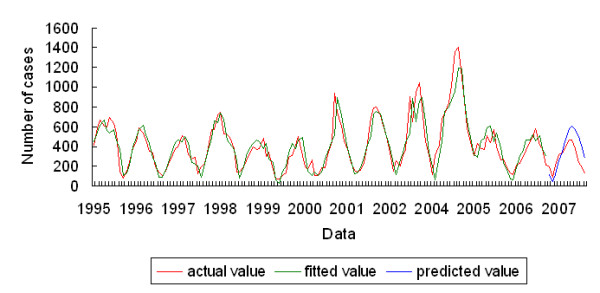
**Fitted, predicted and actual numbers of malaria cases in Hainan, China, 1995-2007**. The *F *of the model fitting was 85.06 (*P *< 0.05), and the adjusted *R *^2 ^was 0.81. The fitted and predicted values were close to the actual value (*R *^2 ^= 0.70).

The model effectively explained the association between the malaria epidemics and climate factors (Figure 5, model fitting *F *= 85.06, *P *< 0.05, adjusted *R *^2 ^of fitting = 0.81). Moreover, the autocorrelations of the fitting residuals were not significant (*P *> 0.05, Table 2), indicating that the model extracted the information sufficiently. This model was used to predict the number of malaria cases in each month in 2007, assuming that other factors were unchanged. There was no significant difference between the monthly predicted value and the actual value (*t *= -1.91, *P *= 0.08). *R *^2 ^for predicting was 0.70 and autocorrelations of predictive residuals were not significant (*P *> 0.05, Table 2), indicating that the model had a good predictive ability.

## Discussion

The results of this study confirmed that the spatiotemporal distribution of malaria in Hainan varied in different areas and during different years, which is consistent with the results of Wen et al. and Su et al. [11,21]. These findings indicate that public health resource allocations should focus on the areas and months with the highest malaria risk in Hainan.

Previous studies have debated the association between malaria epidemics and temperature in areas severely affected by malaria around the world [13,19,24,25] as well as in many areas of China [15,26-30]. A number of researchers suggested that temperature might be a major determinant of malaria epidemics in China; however, the temperature indices (mean temperature, mean maximum temperature and mean minimum temperature) that played the most important role varied in different areas [15,26-28]. In contrast, some researchers reported that the association between malaria epidemics and temperature was not significant [29] or that it was uncertain and depended on the time scales [30]. The uncertainty of weather predictors makes it difficult to predict malaria epidemics effectively and target measures for malaria control and prevention in Hainan. The results of this study showed that both the mean temperature of the previous month (MT _1_) and the mean temperature of the previous two months (MT _2_) were positively associated with malaria incidence in Hainan. Therefore, malaria epidemics can be accurately predicted by monitoring the fluctuations of MT _1 _and MT _2 _in the area, and targeted countermeasures can be taken ahead of time. These strategies will make malaria surveillance and control in Hainan more effective.

Accumulative rainfall (R) was not included in the model as a predictor of malaria epidemics although it was correlated with malaria incidence. This finding was not consistent with previous findings from field studies in China [15,27,28]. The absence of R in the model may be attributed to the co-linearity between R and other factors, which were incorporated in the model. In addition, mean relative humidity (MH) was not related to malaria incidence in Hainan. The high, stable daily MH of approximately 80% [21] is suitable for malaria transmission, but fails to explain the distinctive fluctuations in malaria incidence in Hainan. These results indicate that it is not necessary to consider R and MH to make malaria epidemic predictions in Hainan, which makes malaria surveillance simpler in this area.

Studies performed by Wen et al. [21] constructed a linear model using climate factors in Hainan to predict malaria epidemics (adjusted *R *^2 ^of fitting = 0.72). This model would have been more effective if it had evaluated the fitting residual and predictive validity. Furthermore, it is questionable whether a simple linear model can be used to predict the association between malaria epidemics and climate factors effectively. In this study, a model was constructed to predict malaria epidemics in Hainan by incorporating climate variables of previous months and employing the power function to describe the relationship. This model was reliable and had a good fit and predictive validity (adjusted *R *^2 ^of fitting = 0.81, *R *^2 ^of predicting = 0.70). In addition, this model was constructed using relatively long-term data from a twelve-year period, making the results more reliable than the previous report, which incorporated data from six years. Therefore, this model will provide more accurate and useful information for malaria surveillance in Hainan.

It should be noted that this study examined only climate factors and auto regression on malaria epidemics, without taking socioeconomic circumstances and countermeasures into account. The *R *^2 ^for predicting was less than that of fitting, indicating the association between malaria epidemics and climate factors may be not stable. However, the climate provides the framework for malaria transmission, and other factors can only affect malaria transmission when it is climatically suitable [31]. Therefore, notwithstanding this limitation, the present study demonstrates that MT _1_, MT _2 _and N _1 _can be used to fit and predict malaria epidemics in Hainan. These results lay a foundation for malaria surveillance and control in this area. As a next step, it will be advantageous to explore the method for assigning fluctuant coefficients to climate factors to improve the predictive validity of the model further.

## Conclusions

The spatiotemporal distribution of malaria in Hainan varied in different areas and during different years. The good fitting and predictive validity of the multivariate time series model indicated that MT _1_, MT _2 _and N _1 _can be used to fit and predict malaria epidemics in Hainan. These findings will simplify malaria surveillance and will help make the control of malaria more targeted in Hainan.

## Abbreviations

MT is the mean temperature of each month. MT _0, _MT _1, _MT _2 _and MT _3 _represent the mean temperature of current month, of the previous month, of the previous two months and of the previous three months, respectively. MaxT is the mean maximum temperature of each month. MaxT _0, _MaxT _1, _MaxT _2 _and MaxT _3 _represent the mean maximum temperature of the current month, of the previous month, of the previous two months and of the previous three months, respectively. MinT is the mean minimum temperature of each month. MinT _0, _MinT _1, _MinT _2 _and MinT _3 _represent the mean minimum temperature of the current month, of the previous month, of the previous two months and of the previous three months, respectively. R is the accumulative rainfall of each month. R _0, _R _1, _and R _2 _represent the accumulative rainfall of the current month, of the previous month and of the previous two months, respectively. MH is the mean relative humidity of each month. N _1, _N _2, _and N _3 _represent the number of cases of the previous month, of the previous two months and of the previous three months, respectively.

## Competing interests

The authors declare that they have no competing interests.

## Authors' contributions

DX, YL and SW were involved in the research design, execution and write-up of the first draft of the manuscript. LF, SW, DX, GW and LL contributed to data collection and data analysis. SW, YY, DZ and WC all advised on the design of the study, and the analysis and interpretation of the results. All authors read and approved the final manuscript.
